# A Plant Extract Acts Both as a Resistance Inducer and an Oomycide Against Grapevine Downy Mildew

**DOI:** 10.3389/fpls.2018.01085

**Published:** 2018-07-25

**Authors:** Yuko Krzyzaniak, Sophie Trouvelot, Jonathan Negrel, Stéphanie Cluzet, Josep Valls, Tristan Richard, Ambrine Bougaud, Lucile Jacquens, Agnès Klinguer, Annick Chiltz, Marielle Adrian, Marie-Claire Héloir

**Affiliations:** ^1^UMR 1347 Agroécologie, AgroSup Dijon, Centre National de la Recherche Scientifique, Institut National de la Recherche Agronomique, Université Bourgogne Franche-Comté, Dijon, France; ^2^Université de Bordeaux, Institut des Sciences de la Vigne et du Vin, EA 4577, Institut National de la Recherche Agronomique, USC 1366, Unité de Recherche Œnologie, Villenave d’Ornon, France

**Keywords:** biocontrol, *Vitis vinifera*, *Plasmopara viticola*, induced resistance, plant protection, oomycide, elicitor

## Abstract

Protecting vineyards from cryptogamic diseases such as downy mildew, caused by *Plasmopara viticola*, generally requires a massive use of phytochemicals. However, the issues on unintentional secondary effects on environment and human health, and the occurrence of *P. viticola* resistant strains, are leading to the development of alternative strategies, such as the use of biocontrol products. In this paper, we evidenced the ability of a plant extract to protect grapevine from *P. viticola*. Further experiments carried out both on cell suspensions and on plants revealed that plant extract activates typical defense-related responses such as the production of H_2_O_2_, the up-regulation of genes encoding pathogenesis-related proteins and stilbene synthase, as well as the accumulation of resveratrol or its derivative piceid. We also brought to light a strong direct effect of PE on the release and motility of *P. viticola* zoospores. Furthermore, we found out that PE application left dried residues on leaf surface, impairing zoospores to reach stomata. Altogether, our results highlight the different modes of action of a new biocontrol product able to protect grapevine against downy mildew.

## Introduction

Although wine industry has been meeting an economic, social and cultural wealth, it is currently facing multiple difficulties. Indeed, adapting to climate changes and keeping on protecting vineyards from major diseases are a worldwide challenging concern. Among cryptogamic diseases, downy mildew can cause severe reductions of the yields, especially in vineyards with rainy springs (Gessler et al., 2011). This disease is caused by the biotrophic oomycete *Plasmopara viticola* [(Berk. and Curt.) Berl. and De Toni], which infects all green parts of the plant. During *P. viticola*’s asexual cycle, motile zoospores released from sporangia reach stomata where they encyst. The pathogen then develops an intercellular mycelium with haustoria for nutrient uptake from mesophyll cells. It next emits sporangiophores from other stomata to sporulate, which constitute a source of inoculum for next infections. Generally, to ensure the quantity and the quality of the harvest, the management of downy mildew and cryptogamic diseases requires a massive use of fungicides. However, some of them cause unintentional secondary effects on environment and human health (Gill and Garg, 2014). Therefore, several actors, including researchers and farmers, now focus on using alternative or complementary strategies, such as the use of biocontrol products (Gessler et al., 2011).

Among biocontrol products, crude extracts of plants are widely studied, since they can contain a rich cocktail of active compounds. They can contain alkaloids, phenolic compounds or essential oils, which can exhibit direct toxicity toward plant pathogens. Abundant literature is available about antifungal activity of plant extracts (PEs; for review, see Soković et al., 2013; Shuping and Eloff, 2017). For example, weed extracts showed strong antifungal activity against *Fusarium oxysporum* *in vitro* (Sharma and Kumar, 2009). Leaf extracts and essential oils of *Cymbopogon citratus* or *Eucalyptus citriodora* inhibited the mycelial growth of *Didymella bryoniae*, the causal agent of gummy stem blight on *Cucurbitaceae* (Fiori et al., 2000). Considering grapevine protection, extracts from *Vitis* canes (Schnee et al., 2013), *Inula viscosa* oily paste extracts (Cohen et al., 2006) or neem oil extract (Achimu and Schlösser, 1992) exhibited direct toxicity against *P. viticola*. Crude extracts of plants can also contain other compounds such as polysaccharides, acting as elicitors, able to activate defense mechanisms in host plants (Ebel and Cosio, 1994).

Plant perception of elicitors leads to a cascade of signaling events including: ions fluxes, plasma membrane depolarization (Lecourieux et al., 2002; Vandelle et al., 2006), nitric oxide and reactive oxygen species (ROS, among them H_2_O_2_) production, acting as secondary messengers (Garcia-Brugger et al., 2006). Moreover, a phosphorylation cascade of MAPKs is activated. All this complex cascade of signaling events induces the expression of defense genes. This leads to the synthesis of (i) pathogenesis-related (PR) proteins, including hydrolytic enzymes (e.g., β-1,3-glucanases and/or chitinases), which degrade pathogen cell wall (van Loon et al., 2006); (ii) antimicrobial compounds, such as phytoalexins (Adrian et al., 2012); (iii) compounds involved in the cell wall reinforcement (Underwood, 2012), like callose deposits or hydroxyproline rich glycoproteins (HRGPs). These defense responses are regulated by phytohormones, such as salicylic acid (SA), jasmonic acid (JA) and ethylene (Pieterse et al., 2009).

In grapevine, purified molecules or natural extracts able to induce defense reactions have been widely reported (for review Delaunois et al., 2014). Nonetheless, few of them lead to an actual resistance against pathogens, especially in vineyard conditions (Walters et al., 2013; Delaunois et al., 2014; Trouvelot et al., 2014). In this context, biocontrol products are of interest as they can activate defenses and/or exhibit direct toxicity against pathogen. The aim of this study is to evaluate the efficacy of a novel formulated PE, and to understand its mode(s) of action in grapevine/*P. viticola* interaction. Firstly, PE’s efficacy against downy mildew was evaluated on plants. Secondly, eliciting properties of PE were investigated by analyzing defense responses on grapevine cell suspensions and plants. Finally, direct toxic effects of PE on *P. viticola* were assessed on the release and motility of zoospores, and the early infection steps.

## Materials and Methods

For all experiments, three independent biological repetitions were performed, unless otherwise specified.

### Compound Preparation for Grapevine Treatment

A PE, was supplied by Arysta Lifesciences-Laboratoires Goëmar (Saint-Malo, France). The origin and the chemical specifications of PE are confidential. The stock solution (80 g L^−1^ of PE) was diluted for experiments in MilliQ^®^ water. PE is formulated in a mixture of two conserving agents (cons. agents) of confidential nature. As control, this mixture was also tested alone for all experiments using a mixture stock solution prepared according to the proportion present in PE stock solution. Equal volume of water was also used as control.

For *in vitro* experiments, we tested concentrations ranging from 0.5 to 3 g L^−1^ PE to find a non-lethal dose for grapevine cells at 24 h post-treatment (hpt), as described in the section “Viability Assessment.” Then, this PE concentration was used for all analyses. For *in planta* experiments, the concentration used was 5 g L^−1^ PE, as recommended by the providing company.

### Cell Suspension Culture, Sampling, and Viability Assessment

#### Cell Culture and Preparation

Grapevine (*Vitis vinifera* L. cv. Gamay) cell suspensions were cultivated in Nitsch–Nitsch medium (Nitsch and Nitsch, 1969) on a rotary shaker (125 rpm) at 25°C and under continuous light. They were subcultured every 7 days by transferring 10 mL of cell suspensions into 90 mL of new culture medium. Seven-day-old cultures were diluted twice in Nitsch–Nitsch medium 24 h prior to use for all experiments.

For early signaling events (ROS and MAPK), cells were washed twice with the equilibration buffer M10 (10 mM MES, 175 mM mannitol, 0.5 mM K_2_SO_4_, 0.5 mM CaCl_2_; pH 5.3), then re-suspended at 0.1 g fresh weight of cells (FWC) per mL in M10, and equilibrated for 2 h under light (25°C, 125 rpm), before treatments. For gene expression and resveratrol analyses, cells were adjusted at 0.1 g FWC mL^−1^ in Nitsch–Nitsch medium before treatments.

#### Sampling

For MAPK and gene expression analyses, aliquots of treated cell suspensions (1.5 and 2 mL, respectively) were separated from culture medium through vacuum filtration on GF/A Whatman^TM^ filters. Cells were collected into 2 mL-microtubes containing a 6 mm glass bead, then frozen in liquid nitrogen and kept at −80°C before analysis. Samples were ground with a mixer mill twice for 30 s at 30 Hz (MM 200, Retsch) before extraction. For resveratrol analyses, samples were collected simultaneously as those intended for gene expression, with a 5 mL-microtube placed beneath the filter, to recover 2 mL of corresponding culture medium, then kept at −20°C before analysis.

#### Viability Assessment

Fluorescein diacetate (FDA), a cell permeant esterase substrate, was used to assess cell viability. Twenty microliters FDA (500 μg mL^−1^) were added to 1 mL of cell suspension at 24 hpt. To visualize living cells, observations were immediately realized using an epifluorescence microscope [Leica, λ_exc_ 450–490 nm, λ_em_ 515 nm (filter LP), magnification ×400] equipped with a digital camera. Ten pictures were acquired per condition, and percentage of viability was assessed extemporaneously by counting living and dead cells on each field of observation.

### Plant Production, Treatment, and Inoculation

*Vitis vinifera* L. cv. Marselan plants, susceptible to *P. viticola*, were obtained from herbaceous cuttings placed in individual pots (8 cm × 8 cm × 8 cm) containing a mixture of peat and perlite (3/2, v/v). They were grown until 5-leaf stage at 23°C/15°C (day/night), under a 16-h light photoperiod in a greenhouse. Plants were sub-irrigated with a nutritive solution (N/P/K 10-10-10, Plantin, France).

PE, cons. agents or water (as controls) were sprayed onto upper and lower leaf faces of the second and third youngest fully expanded leaves, until run-off point (Steimetz et al., 2012). Unless otherwise specified, 48 hpt-treated leaves were inoculated with *P. viticola* by spraying a freshly prepared sporangia suspension at 2.10^4^ sporangia mL^−1^ (Kim Khiook et al., 2013).

### Protection Assays Against *P. viticola*

PE efficacy against *P. viticola* was assessed as described by Kim Khiook et al. (2013). Briefly, 6 days after inoculation, leaf disks were punched out and placed with the abaxial side uppermost, on a moist Whatman paper, in a closed plastic box. This system was left overnight in darkness and saturated relative humidity to trigger sporulation. Disease intensity was assessed by measuring the leaf area covered by the pathogen sporulation using a “macro” developed for the image analysis Visilog 6.9 software (Noesis, France; Kim Khiook et al., 2013). Forty leaf disks per condition were used and four independent biological repetitions were performed.

### Defense-Related Responses Assessment

#### H_2_O_2_ Production

H_2_O_2_ production was measured in cell suspension as described by Gauthier et al. (2014). Measurements were carried out by using a luminol chemiluminescence assay with a luminometer (Lumat LB 9507, Berthold, Evry, France). Two hundred and fifty microliters of cell suspension were added to 300 μL of H50 medium (50 mM HEPES, 175 mM mannitol, 5 mM CaCl_2_, 0.5 mM K_2_SO_4_; pH 8.5), and 50 μL of 0.3 mM luminol. Relative luminescence was recorded within a 10 s period and was converted in nmol of H_2_O_2_ per gram of FWC, after the establishment of a H_2_O_2_ reference range with untreated cell suspension.

#### Detection of Phosphorylated MAPKs by Western Blot Analyses

Protein extraction and western blot analyses were performed as described by Poinssot et al. (2003). Briefly, aliquots of treated cells were collected, as described above, at 0, 15, 30, 60 min post-treatment (pt). After protein extraction, aliquots containing 15 μg of protein per sample were solubilized in Laemmli buffer (Laemmli, 1970) and submitted to 10% SDS-PAGE, before transfer to nitrocellulose membrane (Hybond ECL, Amersham biosciences, Munchen, Germany) for western blotting. Phosphorylated MAPKs were detected with an antibody raised against a synthetic phosphor-thr202/tyr204 peptide of human phosphorylated extracellular regulated protein kinase 1/2 (α-perk1/2, Cell Signaling, Danvers, MA, United States). Probing and detection were carried out by an ECL western detection kit (Amersham biosciences, Little Chalfont, United Kingdom).

#### Gene Expression Analyses by qRT-PCR

##### In cell suspension

Aliquots of treated cells were collected as described above at 0, 1, 3, 6, 9, 12, and 24 hpt. Total RNA isolation was carried out with Trizol (Ambion Life technologies, Saint Aubin, France) according to the manufacturer’s instructions. The RNA yield and purity were determined by Nanodrop 2000 (Thermo Scientific, Waltham, MA, United States), then checked on 1% agarose gel. Total RNA (1 μg) was used to synthesize cDNA using Superscript III reverse transcriptase kit (Life technologies, Saint Aubin, France). qRT-PCR experiments were performed using the Absolute^TM^ qPCR Sybr Green ROX mix (Thermo Scientific, Waltham, MA, United States) as previously described by Gamm et al. (2011).

##### In foliar tissues

Treated leaves were collected [0, 24, 48 hpt and at 24, 48 h post-inoculation (hpi)] and immediately frozen in liquid nitrogen. Total RNA was extracted from 100 mg of fine ground leaves with Purelink^TM^ Plant RNA Reagent (12322-012, Invitrogen, Winooski, VT, United States) according to the manufacturer’s instructions, with an extra step with chloroform to obtain clear aqueous phase. DNA contaminations were removed with the DNA-free^TM^ DNA removal kit (AM1906, Ambion Life Technologies, Saint Aubin, France) according to the manufacturers’ specifications. Concentration and purity of RNA, reverse transcription and qRT-PCR steps were performed as for cell suspension study.

For all experiments, the relative change of defense gene expression was determined with the 2^−ΔΔCT^ method (Livak and Schmittgen, 2001). Reference genes, *EF1α* or *EF1γ*, for cell and plant assays, respectively, were used as internal control (Gamm et al., 2011; Dufour et al., 2013). Sequences of the primer pairs used are reported in **Supplementary Table S1**.

#### Stilbene Analyses

##### Quantification of resveratrol in cell suspensions

Aliquots of culture medium were collected as described above at 0, 1, 3, 6, 9, 12, and 24 hpt. *Trans*-resveratrol was analyzed by RP-HPLC by using a Beckman System Gold chromatography system equipped with a diode array detector Model 168 and a Beckman 507 sample injector equipped with a 20 μL sample loop, as described by Krzyzaniak et al. (2018).

##### Quantification of stilbenes in leaf tissues

Samples collected for defense-related gene expression analysis were also used for stilbene analyses. One milliliter of methanol was added to freeze-dried leaf powder (80 mg) and put into an ultrasonic water bath at 25°C for 15 min. After centrifugation (20,000 × *g*, 5 min), the supernatant was removed and conserved in a new tube. The powder was extracted again four more times as previously described. All the supernatants were evaporated to a 500 μL final volume to which 4.5 mL of water were added into a volumetric flask. The diluted phenolic extracts were centrifuged at 4500 × *g* for 5 min.

Analysis of stilbenes was performed by a 1260 Infinity UPLC (Agilent Technologies, Courtaboeuf, France) coupled to a 6430 triple quadrupole mass spectrometer (Agilent Technologies, Courtaboeuf, France), equipped with a Gerstel MPS2 autosampler. Five microliters of leaf extract were injected into an Agilent Zorbax SB-C18 (100 mm × 2.1 mm, 1.8 μm) thermostated at 40°C, and separation of the compounds was performed at a flow rate of 0.4 mL min^−1^ with a mobile phase composed of solvent A (distilled water, 0.1% formic acid) and solvent B (acetonitrile, 0.1% formic acid). The run was as follows: 0 to 3.5 min, 18% B; 3.5 to 6.5 min from 18% B to 33% B; 6.5 to 12 min from 33% B to 40% B; 12 to 13 min 40% B to 95% B; 13 to 16 min, 95% B; 16 to 16.5 min, from 95% B to 18% B. Total ion chromatograms were obtained using negative mode. The parameters were: capillary voltage, +3 kV; nebulizer pressure, 15 psi; dry gas, 11 L min^−1^; dry temperature, 350°C. Specific MRM transitions for each stilbene were used for identification and quantification with the Agilent Data Analysis software (Agilent Technologies, Courtaboeuf, France). Stilbenes (*trans*-resveratrol, piceid, piceatannol, astringin, α-viniferin, and miyabenol) were determined from calibration curves of pure standards (injected concentrations ranging from 0.004 to 10 μg mL^−1^) and concentrations were expressed in μg g^−1^ of pure phenolic compound. The linearity of the response of the standard molecules was checked by plotting the peak area versus the concentration of the compounds. All the standard stilbenes were produced and purified in laboratory conditions (UR Oenology, Villenave d’Ornon, France).

### Evaluation of PE Toxicity Toward *P. viticola*

#### *In Vitro* Experiments

To assess the effect of treatments on the ability of *P. viticola* sporangia to release zoospores, 5 mL of sporangia suspension (10^5^ sporangia mL^−1^) were incubated with PE (0.5 to 2.5 g L^−1^), cons. agents or water in glass erlenmeyer for 2 h at room temperature, and then mounted in Malassez hemacytometer. The number of swimming zoospores crossing a defined unit square for 1 min were counted under light microscope (magnification ×100, Leica DME).

To assess the effect of treatments on the motility of released zoospores, untreated sporangia suspension was let for 2 h, time necessary for zoospore release. After checking their motility, zoospores were treated by PE (0.5 g L^−1^), cons. agents or water, and swimming zoospores were counted 2 min later as described above.

#### *In Planta* Experiments

In order to evaluate the direct effect of PE on *P. viticola* development *in planta*, plants were inoculated (10^5^ sporangia mL^−1^) after only 2 hpt, instead of 48 hpt, a time period supposed to be insufficient for the plant to activate its full defense responses (Trouvelot et al., 2008). At 24 and 48 hpi, leaf disks were collected, clarified, observed and analyzed as described by Kim Khiook et al. (2013). Pathogen presence was detected after aniline blue staining, and observation by epifluorescence microscopy [magnification ×200, Leica, λ_exc_ 340–380 nm, λ_em_ 425 nm (LP filter)]. In that context, pathogen presence appeared in light blue fluorescence. For 24 hpi samples, the number of stomatal infection sites per field of observation was counted and for 48 hpi samples, internal colonization by *P. viticola* was evaluated by image analysis using Visilog software. Three pictures per disk were acquired from 10 disks per biological repetition per condition.

#### Observation of Leaf Surface by Scanning Electron Microscopy (SEM)

At 24 hpi, 0.5 cm^2^ leaf squares were excised from treated and inoculated (10^5^ sporangia mL^−1^) leaves. The leaf surface was then characterized by cryo-scanning electron micrographs (cryo-SEMs) taken using a Hitachi (SU 8230) scanning electron microscope equipped with Quroum PP3000 t cryo attachment. Prior to imaging, leaf samples were rapidly frozen in liquid nitrogen in order to fix them. Thereafter they were sublimated at −90°C for 5 min and coated with a thin platinum layer by sputtering at 5 mA for 10 s.

## Results

### PE Protects Grapevine Leaves Against *P. viticola*

In order to check the ability of PE to protect grapevine against *P. viticola*, plants were sprayed with PE solution at 5 g L^−1^ as recommended by the providing company, then inoculated with *P. viticola* 48 h later.

**FIGURE 1:**
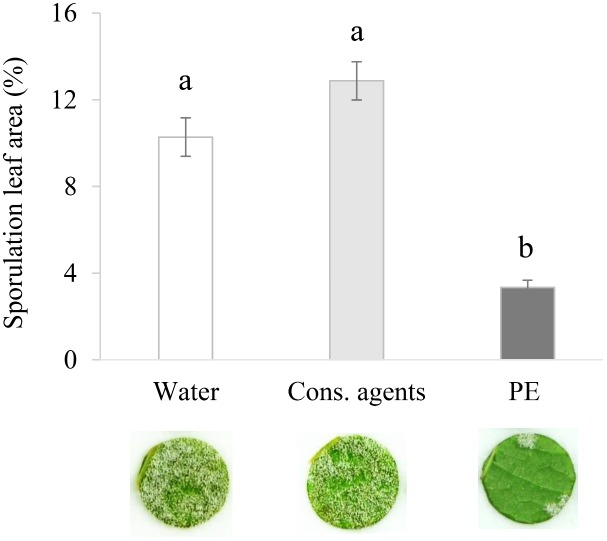
Plant extract (PE) protects grapevine leaves against *P. viticola.* Plants grown in greenhouse conditions were treated by PE at 5 g L^−1^, or equal volumes of conserving (cons.) agents or water, and inoculated at 48 hpt with a suspension of *P. viticola* sporangia (2.10^4^ sporangia mL^−1^). At 6 dpi, 40 disks per condition (i.e., five disks per leaf, with two leaves per plant, and four plants per condition) were punched out. Leaf disk sporulating area was evaluated at 7 dpi by image analysis. Results correspond to the mean ± confidence interval of four independent biological repetitions. Significant differences (*p* ≤ 0.05) were identified with Kruskal–Wallis coupled with Dunn’s multiple comparison test. Conditions with different letters are significantly different.

PE induced a good level of protection since a significant reduction of *P. viticola* sporulation, by 67% or 74% compared to water or cons. agents, respectively, was observed at 6 dpi (**Figure 1**). Compared to water control, cons. agents alone had no significant effect on disease severity.

### PE Activates Defense Responses in Grapevine Cell Suspensions

We first looked into PE ability to trigger some defense markers in cell suspensions. To determine the concentration to use on cell suspension, viability test after FDA staining was performed at 24 hpt according to a PE concentration range. PE reduced the percentage of cell viability at all assessed concentrations (by 20% and 40% from 1 to 3 g L^−1^), except at 0.5 g L^−1^ (**Supplementary Figure S1**), which was therefore chosen for the following experiments. Cons. agents, applied at the equal volume of the PE highest concentration, did not induce cell death.

#### PE Induces H_2_O_2_ Production and MAPK Phosphorylation

**FIGURE 2:**
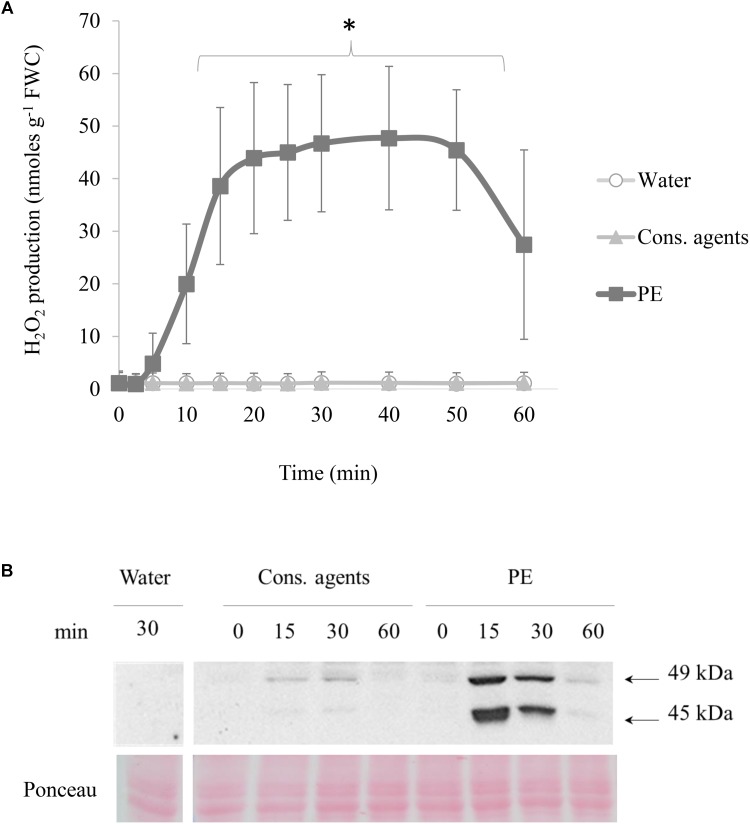
Effects of PE on early signaling events in cell suspensions. Grapevine cell suspensions, adjusted to 0.1 g of fresh weight cell (FWC) mL^−1^, were treated with PE (0.5 g L^−1^), or equal volumes of cons. agents or water. **(A)** H_2_O_2_ production in cell suspension, measured by luminol chemiluminescence method. Data were converted from Relative Luminescence Unit (RLU) to nmoles H_2_O_2_ g^−1^ FWC according to a calibration curve of H_2_O_2_ concentration range. Results correspond to the mean ± confidence interval of three independent biological repetitions. Significant differences were identified with Student *t*-test comparison tests against water condition and conserving agents (^∗^*p* < 0.05). **(B)** Detection of phosphorylated MAPKs by western blotting in grapevine cell suspension. Results show a representative experiment out of three independent biological repetitions. Homogeneous transfer and protein loading were checked with Ponceau staining.

Production of H_2_O_2_ was determined by chemiluminescence assay (**Figure 2A**). In response to PE treatment, cells generated H_2_O_2_ from 5 min pt, and the maximum (about 47 nmol g^−1^ FWC) was reached 20 min after treatment. This level remained steady during 30 min, and decreased from 50 min. Equivalent volume of cons. agents or water did not induce any H_2_O_2_ production.

Phosphorylation of MAPK was investigated by western blotting, with polyclonal antibodies binding specifically to the phosphorylated form of plant ERK-related activated MAPKs (**Figure 2B**). Bands corresponding to two phosphorylated MAPK of 49 and 45 kDa were detected in PE-treated cells. The activation of these MAPK peaked at 15 min pt, was still important at 30 min and decreased to a basal level within 60 min. Only a slight signal was detected in cons. agents-treated conditions especially for 49 kDa MAPK, compared to water control.

#### PE Induces Defense Gene Expression

**FIGURE 3:**
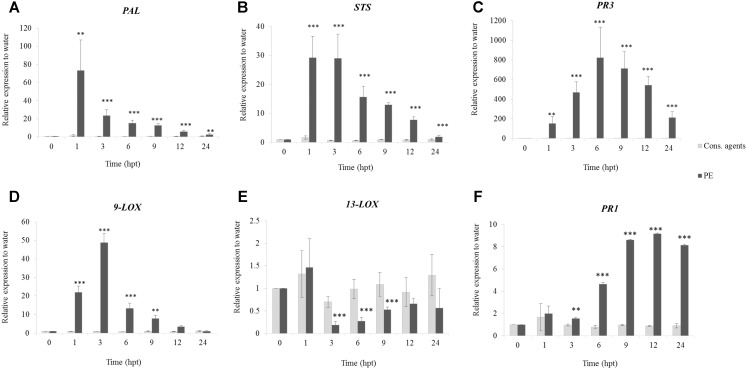
Relative expression of defense-related genes in grapevine cells in response to PE treatment. Cell suspensions, adjusted to 0.1 g FWC mL^−1^, were treated with PE (0.5 g L^−1^), or equal volumes of cons. agents or water. Six genes encoding a **(A)** phenylalanine ammonia-lyase (PAL); **(B)** stilbene synthase (STS); **(C)** chitinase 4c (PR3); **(D)** lipoxygenase 9 (9-LOX); **(E)** lipoxygenase 13 (13-LOX); and **(F)** pathogenesis-related protein 1 (PR1) were investigated by qRT-PCR. Results represent relative fold expression calculated with the 2^−ΔΔCt^ method, compared to the housekeeping gene *EF1α* and to water control for each respective time point. Data correspond to the mean ± confidence interval of three independent biological repetitions with three technical replicates each. Significant differences were identified with Student *t*-test compared to cons. agents condition (^∗^*p* < 0.05; ^∗∗^*p* < 0.01; ^∗∗∗^*p* < 0.001).

qRT-PCR was carried out under a tight time course, in order to follow the expression of six selected defense-related genes (**Figure 3**) encoding a phenylalanine ammonia-lyase (PAL), a stilbene synthase (STS), a pathogenesis-related protein 1 (PR1), two lipoxygenases (9-LOX, 13-LOX) and a chitinase 4c (PR3). Globally, expression of all genes was induced in response to PE treatment with differences in kinetics and intensities, except for *13*-*LOX*, which was repressed. *PAL* (**Figure 3A**) and *STS* (**Figure 3B**) were early upregulated, peaking from 1 hpt with 70-fold and 30-fold relative expression to water, respectively, before decreasing. Interestingly, *9-LOX* was upregulated 50-fold at 3 hpt (**Figure 3D**), while *13-LOX* was downregulated (0.19-fold) at the same time point (**Figure 3E**). *PR3* was the gene the most upregulated by PE treatment, with an accumulation of transcripts peaking (800-fold) at 6 hpt, before decreasing slowly thereafter (**Figure 3C**). However, at 24 hpt, it still remained quite high at 200-fold. Finally, a progressive upregulation of *PR1* from 6 to 24 hpt was detected with a maximum of ninefold (**Figure 3F**). Results also showed that, cons. agents did not seem to affect the expression of the studied genes.

#### PE Increases Resveratrol Production

**FIGURE 4:**
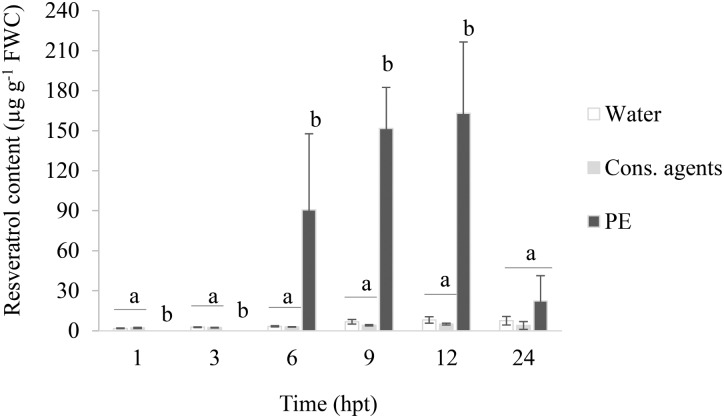
*Trans*-resveratrol accumulation in the culture medium of PE-treated cells. Cell suspensions, adjusted to 0.1 g FWC mL^−1^, were treated with PE (0.5 g L^−1^), or equal volumes of cons. agents or water. The culture medium was collected by vacuum filtration. Quantification of *trans*-resveratrol was performed by HPLC analysis and results correspond to the mean ± confidence interval of three independent biological repetitions. Significant differences (*p* ≤ 0.05) were identified with Kruskal–Wallis coupled with Dunn’s multiple comparison test. Conditions with different letters are significantly different.

The grapevine phytoalexin resveratrol was quantified in the culture medium of grapevine treated cells (**Figure 4**). A basal amount was detected in water and cons. agents-treated conditions along the time course of the experiment, ranging from 2 to 8 μg resveratrol g^−1^ FWC. No significant difference was observed between these two controls. Curiously, in response to PE-treatment, no resveratrol could be detected at 1 and 3 hpt (or under detection threshold). However, its level considerably increased from 6 hpt, peaked at 12 hpt (around 160 μg g^−1^ FWC), and declined back to basal level at 24 hpt.

### PE Also Activates Defense Responses *in Planta*

#### PE Induces Defense Gene Expression

**FIGURE 5:**
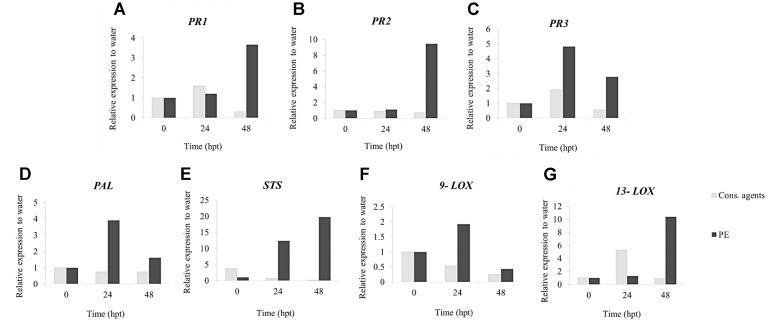
Relative expression of defense-related genes in PE-treated grapevine leaves. Plants were treated with PE (5 g L^−1^), equal volumes of cons. agents or water and leaves were collected at 24 and 48 hpt. Six genes encoding a **(A)** pathogenesis-related protein 1 (PR1); **(B)** β-1.3 glucanase (PR2); **(C)** chitinase 4c (PR3); **(D)** phenylalanine ammonia-lyase 1 (PAL); **(E)** stilbene synthase (STS); **(F)** lipoxygenase 9 (9-LOX); and **(G)** lipoxygenase 13 (13-LOX) were investigated by qRT-PCR. Results represent relative fold expression calculated with the 2^−ΔΔCt^ method, compared to the housekeeping gene *EF1γ* and to water control for each respective time point. Data represent mean from three technical replicates of one representative biological repetition.

In order to complete the results obtained with cell suspensions, qRT-PCR analyses of the same defense-related genes were conducted *in planta* in response to treatments before and after inoculation. In addition, the expression of *PR2* encoding a β-1,3-glucanase, a lytic enzyme toward oomycete cell walls, was also followed. For samples taken before inoculation, results showed that, conversely to cons. agents, PE globally induced all studied gene expressions (**Figure 5**). The upregulation of *PR3*, *PAL*, and *9-LOX* (**Figures 5C,D,F**), was the most intense at 24 hpt whereas the expression of *PR1*, *PR2*, *STS*, and *13-LOX* (**Figures 5A,B,E,G**) peaked at 48 hpt. For inoculated samples, a strong variability was observed among biological repetitions, and results were too variable to be interpreted (data not shown).

#### Among Stilbene Compounds, PE Induces Only Piceid Production

Stilbene compounds (*trans*-resveratrol, piceid, piceatannol, astringin, α-viniferin and miyabenol) were quantified by UPLC. Among the six analyzed compounds, only piceid was significantly detected whatever the conditions.

**FIGURE 6:**
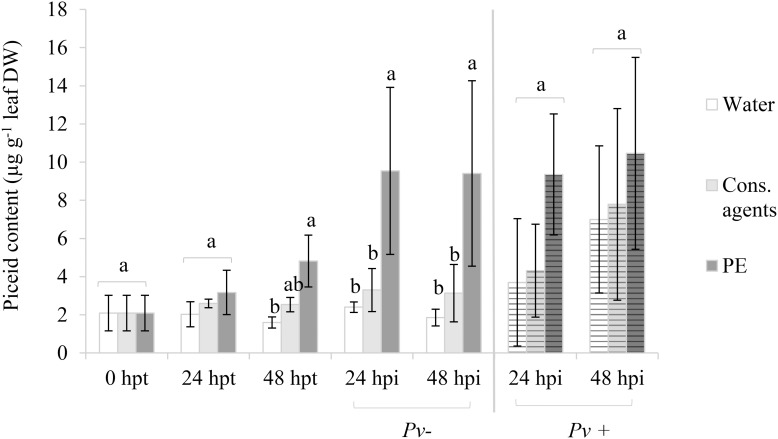
Piceid accumulation *in planta* in response to PE treatment with or without *P. viticola* inoculation. Plants were treated with 5 g L^−1^ PE, or equal volumes of cons. agents or water, and mock (Pv–) or *P. viticola* (Pv+) inoculated at 48 hpt. Leaves were collected at 24 and 48 hpt, and at 24 and 48 hpi. Piceid was analyzed by UPLC. Results correspond to the mean ± standard deviation of three independent biological repetitions. Significant differences (*p* ≤ 0.05) were identified with Kruskal–Wallis coupled with Dunn’s multiple comparison test. Conditions with different letters are significantly different.

Without pathogen challenge (**Figure 6**, unstriped bars), in the water control, a basal level of about 2 μg piceid g^−1^ DW was found through the kinetic. In cons. agents treated samples, piceid tended to accumulate but it was not statistically different from the water control. Conversely, PE induced a progressive and significant increase of piceid production, mainly after 48 hpt as observed in mock-inoculated samples (*Pv*–). Indeed, at 24 hpi (i.e., 72 hpt) or 48 hpi (i.e., 96 hpt) mock-inoculated, piceid content was twofold higher in PE condition than in controls (10.5 and 3 μg g^−1^ DW, respectively).

Results also showed that *P. viticola* inoculation itself increased piceid production (**Figure 6**, striped bars). Indeed, piceid content in water control was four times higher at 48 hpi than before pathogen challenge at 48 hpt. Though, a great variability between biological repetitions was observed after inoculation, and no significant difference between treatments was found.

### PE Inhibits *P. viticola* Zoospores Release, Motility, and Infection Process

#### Inhibition of Zoospore Release and Motility *in Vitro*

**FIGURE 7:**
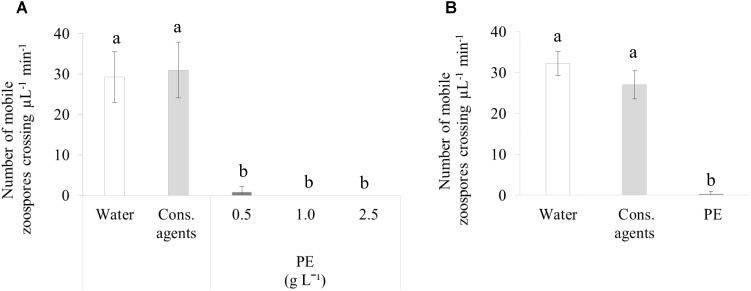
*In vitro* assessment of direct effect of PE on *P. viticola*. **(A)** Release of zoospores from sporangia at 2 hpt. **(B)** Motility of zoospores already released at 2 min post treatment. Sporangia were collected from a sporulating leaf and concentrated at 10^5^ sporangia mL^−1^, then PE was applied at different concentrations. As controls, cons. agents or water were added (volumes corresponding to the highest concentration of PE). Motile zoospores crossing one unit of Malassez hemacytometer were counted during 1 min. Results correspond to the mean ± confidence interval of three independent biological repetitions, with three technical repetitions each. Significant differences (*p* ≤ 0.05) were identified with Kruskal–Wallis coupled with Dunn’s multiple comparison test. Conditions with different letters are significantly different.

At 2 hpt, about 30 mobile zoospores μL^−1^ min^−1^ were counted in water or cons. agents-treated sporangia suspension (**Figure 7A**), and no irregular swimming pattern was noticed. Conversely, a stunning failure for sporangia to release their zoospores was observed in response to PE, whatever the concentration tested (0.5, 1, or 2.5 g L^−1^).

We also studied the effect of PE on released zoospores using the lowest dose (0.5 g L^−1^; **Figure 7B**). PE completely stopped the motility of zoospores. Cons. agents seemed to reduce slightly the motility, but there was no significant difference compared to water.

#### Inhibition of Early Infection Steps of *P. viticola in Planta*

**FIGURE 8:**
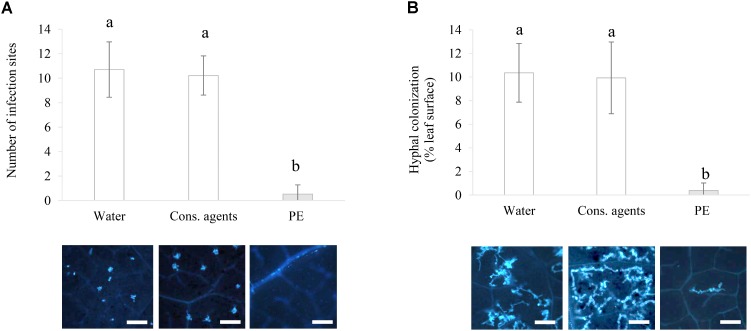
Assessment of direct effect of PE on the early steps of *P. viticola* infection *in planta*. Plants were treated with 5 g L^−1^ PE, or equal volumes of conserving agents or water, and were inoculated with *P. viticola* sporangia suspension (10^5^ sporangia mL^−1^) at 2 hpt. Aniline blue staining was performed to visualize: **(A)** the number of infection sites per observation field, counted at 24 hpi; and **(B)** the hyphal colonization surface (%) per observation field at 48 hpi, estimated by image analysis. Results correspond to the mean ± standard deviation of three independent biological repetitions. Significant differences (*p* ≤ 0.05) were identified with Kruskal–Wallis coupled with Dunn’s multiple comparison test. Conditions with different letters are significantly different. Pictures presented hereby are the ones with the closest values to the mean. Scale bars represent 100 μm.

Since *in vitro* studies cannot completely illustrate the interaction between a biotrophic pathogen and its host plant, experiments were also performed *in planta.* PE significantly reduced the number of both infection sites per field of observation at 24 hpi (**Figure 8A**), and the hyphal colonization by 10 times at 48 hpi (**Figure 8B**), as compared to water or cons. agents-treated leaves.

#### PE Droplet Residues May Trouble Zoospore Swimming

**FIGURE 9:**
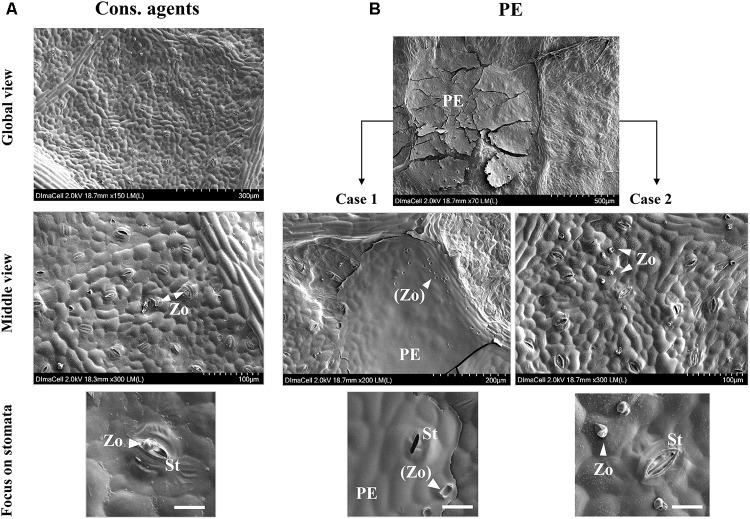
SEM observation of leaf surfaces at 24 hpi after treatment by cons. agents **(A)** or PE **(B)** treatments, and *P. viticola* inoculation (10^5^ sporangia mL^−1^). Top line of pictures show global overview of leaf surface to evidence deposits left on leaf surfaces or not; center line of pictures show middle view in order to point out zoospore presence around potential infection sites. For PE treatments, two cases were observed: (1) dried PE droplet forming a thin layer covering stomata access, or (2), outside of any dried droplet deposit, zoospores not encysted in stomata. Bottom line of pictures show focuses on stomata in each case. PE, PE droplet residue; St, stomata; Zo, zoospore; (Zo), zoospore ‘footprints.’ Embedded scale bars display the corresponding size on pictures: Global view = 300 or 500 μm; Middle view = 100 or 200 μm; Focus on stomata = 20 μm.

Scanning electron microscopy (SEM) observation showed that spray application of cons. agents left no trace on leaf surface (**Figure 9A**, global view). We could note the pathogen encystment in stomata (**Figure 9A**, middle view, see focus on stomata), demonstrating that the infection succeeded. Conversely, PE-treated leaves showed the presence of dried deposits of different sizes on leaf surface, ranging from 50 to 500 μm of diameter (**Figure 9B**, global view). The leaf surface was not completely covered by these deposits, and two cases could be observed. In case 1, presence of PE residues was detected, and several ‘footprints’ looking like *P. viticola* structures, were spotted on the deposit (**Figure 9B**, case 1, middle view, see focus on stomata). In case 2, there was no PE residue, and *P. viticola* structures were observed on the leaf surface (**Figure 9B**, case 2, middle view). Indeed, according to their size, these structures were identified as zoospores, although they seemed to have lost their flagella (**Figure 9B**, case 2, focus on stomata). Most of them were far from stomata, and the closest were not encysted.

## Discussion

This study aimed to value the use of a novel formulated PE as a means to protect grapevine from downy mildew, within the current biocontrol strategy context. Leaf disks sporulation assays enabled us to reveal its great efficacy against *P. viticola*. This encouraged us to better understand its modes of action, by bringing insights to the following questions: does PE induce resistance against *P. viticola*? Does it directly affect the pathogen as well and how? To this purpose, *in vitro* and *in planta* experiments were carried out to answer these questions.

### PE Is an Elicitor of Grapevine Defenses

We first focused on the potential elicitor capacity of PE on grapevine cell suspension and plants, by following a panel of defense markers. As a first step, the cell suspension model allowed us to better guarantee the bioavailability of PE toward the target cells. The effects of PE were investigated on some early signaling defense events. MAPK phosphorylation was assessed as this event plays a key role in response to abiotic or biotic stresses, and elicitors (Meng and Zhang, 2013). Our results showed that PE induced the phosphorylation of two MAPK of 45 and 49 kDa. In a comparable way, a transient phosphorylation of these MAPK was previously reported for grapevine cells in response to various elicitors such as soybean hydrolysates, oligogalacturonides, flagellin or laminarin (Aziz et al., 2003; Dubreuil-Maurizi et al., 2010; Lachhab et al., 2014; Trdá et al., 2014). PE also induced H_2_O_2_ production, which maximum level remained stable for 30 min. This result differs from previous works for which elicitor treatment rather induced a transient peak of H_2_O_2_ (Ménard et al., 2004; Aziz et al., 2007; Gauthier et al., 2014). The rapid decrease of H_2_O_2_ amount is mainly attributed to detoxification by peroxidases or catalases (Anjum et al., 2016), which use H_2_O_2_ as substrate, in order to limit oxidative burst that can lead to cell death (Baker et al., 1995). This could suggest that PE affects this detoxification process. However, we showed that cell viability was not impacted at the PE concentration used.

PE changed the expression of all studied defense-related genes, which is in common with other documented elicitors (Aziz et al., 2007; Lachhab et al., 2014; Dufour et al., 2016). Interestingly, using cell suspensions, we showed that PE simultaneously upregulated the expression of *9-LOX* and repressed *13-LOX* one. Lipoxygenase gene family encodes enzymes involved in the octadecanoid pathway, resulting in the biosynthesis of oxylipins, which constitute potent signaling or antimicrobial compounds (Blée, 2002; Prost et al., 2005; Shah, 2005). Oxylipins are produced upon pathogen challenge or elicitor applications (Weber, 2002; Randoux et al., 2010; Boubakri et al., 2013). Both 9-LOX and 13-LOX use the same substrate α-linolenic acid but they lead to different products 13-LOX is involved in jasmonate biosynthesis, while 9-LOX leads to other oxylipin synthesis (Porta et al., 2002; Wasternack, 2007). The activation of one biosynthesis pathway is therefore in detriment to the other one. Our results reflect this counterbalance. Curiously, both *LOX* genes were upregulated *in planta*. It would be interesting to further investigate these differences between cell and plant responses.

Furthermore, the effect of PE on phytoalexin production was investigated. Phytoalexins are low molecular weight compounds produced and accumulated in response to stresses (Kuć, 1990). In grapevine, phytoalexins are produced by the phenylpropanoid pathway. The enzyme PAL acts upstream and converts phenylalanine into cinnamic acid, and STS downstream synthesizes resveratrol from *p*-coumaroyl-CoA and 3 malonyl-CoA. Resveratrol is the precursor of other stilbenes after chemical modifications such as glycosylation, methoxylation, and di- or polymerization (Bavaresco and Fregoni, 2001; Chong et al., 2009; Jeandet et al., 2010). In cell suspension, it has been shown that resveratrol can be excreted in the culture medium (Adrian et al., 2012). In our study, we showed that PE upregulated *PAL* and *STS* expressions. Moreover, *trans*-resveratrol transiently accumulated in the culture medium just as Lachhab et al. (2014) observed in response to soybean hydrolysates, within the same order of magnitude. Interestingly, no resveratrol was detected within the first 3 h in response to PE while some was detected in controls. As PE also induced an H_2_O_2_ production, resveratrol may have cross-linked to the cell walls *via* peroxidases as previously reported (Adrian et al., 2012).

In leaf samples, only piceid accumulated. The absence of resveratrol was quite unexpected, as it was often reported in grapevine that foliar application of some elicitors, such as oligosaccharides from *B. cinerea* culture extracts (Saigne-Soulard et al., 2015), chitosan (Aziz et al., 2006) or MeJA (Belhadj et al., 2006) were able to induce piceid, and also resveratrol production. In our conditions, neither resveratrol was detected in untreated-infected samples, whereas authors usually report its accumulation in response to *P. viticola* infection (Schmidlin et al., 2008). Vrhovsek et al. (2012) detected some resveratrol at 6 dpi, whereas it was undetectable at 2 dpi. In our case, samples were collected 1 or 2 days after inoculation, probably prior to resveratrol accumulation, so it could be interesting to assess its production at later time points. Additionally, it has been shown that plants susceptible to *P. viticola* infection can accumulate strong amounts of piceid, a glycosylated compound that is less toxic than the aglycon resveratrol; whereas resistant plants rather accumulate resveratrol or even viniferins, which are more active products in terms of antimicrobial capacity (Pezet et al., 2004).

Taken together, our results demonstrate that PE is an elicitor of defense reactions. However, these latter seemed to be more pronounced and less variable in cell suspension than in plants. It could be explained by PE bioavailability, less guaranteed in plants, because penetration of active molecules through leaf cuticle can be held back if not adequately formulated for instance (Paris et al., 2016).

### PE Has Also an Oomycide Activity Against *P. viticola*

Besides its ability to trigger defense responses, PE showed a direct antimicrobial effect toward *P. viticola*. It prevented zoospore release from sporangia, and inhibited their swimming in *in vitro* tests. Similar studies pointing out toxic effect of PEs against *P. viticola*, as for *Juncus effusus* (Thuerig et al., 2016) or pine extracts (Gabaston et al., 2017). Oomycides can have different modes of action, such as the alteration of zoospore energy production. For example, macrotetrolides antibiotics of *Streptomyces* species displayed similar motility impairing activities against *P. viticola*, *Phytophthora capsici*, and *Aphanomyces cochlioides*. They are suspected to enhance zoospore mitochondrial ATPase activity, leading to ATP depletion and impairment in zoospore motility (Islam et al., 2016).

Microscopic observations of *P. viticola* *in planta* or *in vitro* were consistent since PE exerted a direct effect against the oomycete in each case. PE prevented stomatal encystment, thus confirming that zoospores were rendered unable to reach stomata at the surface of PE-treated leaves for most of them. As Tröster et al. (2017) stated accurately, there is a very short phase in the life cycle of an oomycete, when the pathogen is highly vulnerable between hatching from the sporangium and the encystment at stomata. This short phase was described like the Achilles’ heel of the pathogen. SEM observations showed that many patches of PE residues were still present on the leaf surface at 72 hpt. Even the additional input of water brought by inoculation at 48 hpt did not cleanse or totally remove PE residues, suggesting that PE has notable persistence at leaf surface. After inoculation, some *P. viticola* structures are therefore directly in contact with PE, but conversely to *in vitro* experiments, SEM observations showed that sporangia were able to liberate zoospores at the leaf surface. Indeed, this contact could either kill zoospores directly, impair their swimming or physically block access to stomata. Chitosan application was reviewed to form films, which act as physical barriers around the sites of a pathogen attack (El Hadrami et al., 2010; El Guilli et al., 2016), although no supporting pictures with SEM were done. Using SEM to observe phytosanitary products residues (herbicide or insecticide) on leaves is not recent (Hart, 1979; Pedibhotla et al., 1999). However, this is the first time to our knowledge, that residues of a biocontrol product such as PE are observed *in situ*, especially, in presence of the pathogen.

### Implications for Research Studies Disclosing the Modes of Action of Biocontrol Products

Some authors only study the induction of defense responses to explain the resulting protection against a pathogen (Dutsadee and Nunta, 2008; Jaulneau et al., 2011; Ali et al., 2016). For foliar applications, a case in which the pathogen may directly encounter the product, we believe that information concerning direct toxicity should be required. Chitosan is a well-studied elicitor, which can also have a strong antimicrobial effect (El Hadrami et al., 2010; El Guilli et al., 2016). However, to demonstrate these two modes of action, the concentration, the molecular weight and the degree of acetylation of chitosan used were not always the same. Other authors assessed both induction of resistance and antimicrobial effects, but they showed that the protection was only correlated to eliciting activity (Trouvelot et al., 2008; Boubakri et al., 2013; Nesler et al., 2015; Clinckemaillie et al., 2017). Therefore, the double mode of action of compounds or extracts, such as PE’s, is scarcely published. For example, *Rheum palmatum* root extract and *Frangula alnus* bark extract were able to display both direct toxic effect and induction of resistance on grapevine against downy mildew (Godard et al., 2009).

Finally, it is worth underlining the difficulty of quantifying precisely the part of the protection level due to each mode of action (antimicrobial/defense activation), especially against biotrophic pathogens. It would be possible by using mutant plants deprived from their ability to activate their defense response, for example. Apart from that aspect, the two modes of action of PE make it worth developing as a potential biocontrol product, since if one mode of action is ineffective (e.g., application on young organs with low-responsiveness to induced resistance), the other one may take the lead and keep on protecting the host.

## Conclusion

The efficacy of PE against grapevine downy mildew was described thanks to the complementarity of analyses conducted *in vitro* and *in planta*. It clearly revealed that PE acted both as a resistance inducer and an oomycide against *P. viticola*. Further experiments should be performed to widen PE’s efficacy spectrum to other pathogens and host plants, with the aim of integrating this novel product into biocontrol-friendly crop management strategies.

## Author Contributions

MA, M-CH, and ST led the project. YK, JN, AB, M-CH, AK, and AC shared *in vitro* experiments. YK, ST, LJ, JV, TR, and SC performed analyses *in planta*. YK and M-CH wrote the manuscript. MA, SC, and ST revised the manuscript and contributed to interpret data. All authors reviewed and approved the manuscript.

## Conflict of Interest Statement

The authors declare that the research was conducted in the absence of any commercial or financial relationships that could be construed as a potential conflict of interest.

## Supplementary Material

FIGURE S1 Viability of cells after PE treatment. Grapevine cells were treated with PE (0.5–3 g L^−1^), or with cons. agents or water (volumes corresponding to the highest concentration of PE). At 24 hpt, the viability of grapevine cells was assessed after fluorescein diacetate (FDA) staining. Observation of living cells was realized using an epifluorescence microscope [Leica, λ_exc_ 450–490 nm, λ_em_ 515 nm (filter LP), magnification ×400] equipped with a digital camera. Ten pictures were acquired per condition, and percentage of viability was assessed extemporaneously by counting living and dead cells (about 300 cells per repetition). Results correspond to the mean ± standard deviation of three independent biological repetitions. Significant differences (*p* ≤ 0.05) were identified with Kruskal–Wallis coupled with Dunn’s multiple comparison test. Conditions with different letters are significantly different.

TABLE S1 Sequences of the primers used for qRT-PCR analyses.
