# Supplementary figures and images for: A Plant Extract Acts Both as a Resistance Inducer and an Oomycide Against Grapevine Downy Mildew
Source: Front Plant Sci. 2018 Jul 25;9:1085. doi: 10.3389/fpls.2018.01085 (PMC6068391; doi:10.3389/fpls.2018.01085)

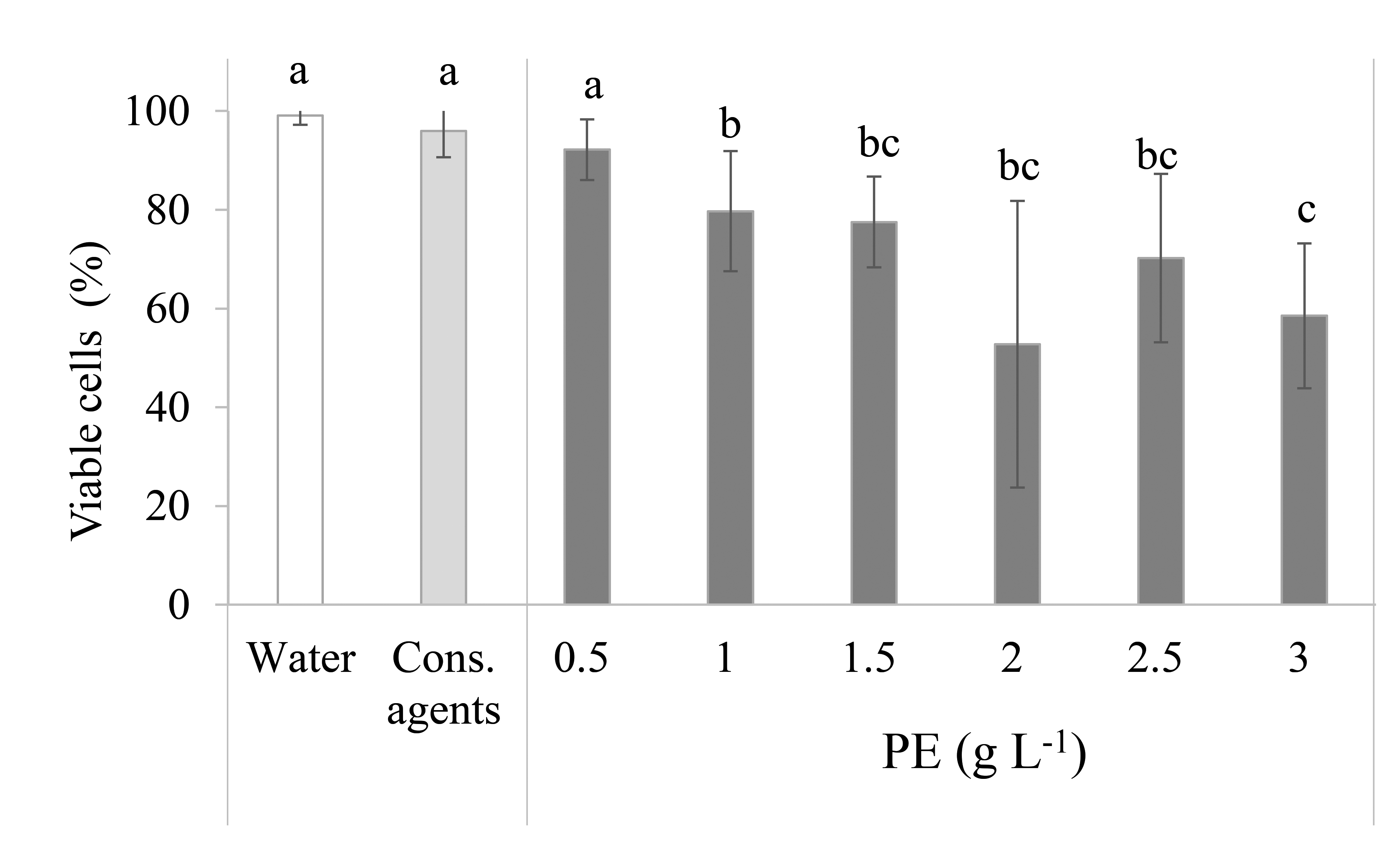

Supplement: FIGURE S1 — Viability of cells after PE treatment. Grapevine cells were treated with PE (0.5–3 g L−1), or with cons. agents or water (volumes corresponding to the highest concentration of PE). At 24 hpt, the viability of grapevine cells was assessed after fluorescein diacetate (FDA) staining. Observation of living cells was realized using an epifluorescence microscope [Leica, λexc 450–490 nm, λem 515 nm (filter LP), magnification ×400] equipped with a digital camera. Ten pictures were acquired per condition, and percentage of viability was assessed extemporaneously by counting living and dead cells (about 300 cells per repetition). Results correspond to the mean ± standard deviation of three independent biological repetitions. Significant differences (p ≤ 0.05) were identified with Kruskal–Wallis coupled with Dunn’s multiple comparison test. Conditions with different letters are significantly different. [file Image_1.TIF]
