# Supplementary material for: A Plant Extract Acts Both as a Resistance Inducer and an Oomycide Against Grapevine Downy Mildew
Source: Front Plant Sci. 2018 Jul 25;9:1085. doi: 10.3389/fpls.2018.01085 (PMC6068391; doi:10.3389/fpls.2018.01085)
Supplement: TABLE S1 — Sequences of the primers used for qRT-PCR analyses. [file Table_1.DOCX]

**Supplemental Table 1.** Sequences of the primers used for qRT-PCR analyses.

| Primers | Sequences | Target genes | References |
| --- | --- | --- | --- |
| Vv_PR1 F  Vv_PR1 R | GCGTGGGTGGGGAATGCCGA  GATGTTGTCCCTGATAGTTGCC | Pathogenesis related 1 | Trdá et al., 2014 |
| Vv_PR2 F  Vv_PR2 R | TCAGCCGTCCTCGGCAAATCA  TTGGCCAGGAGTGGGGAGCC | Glucanase | Lachhab et al.2014 |
| Vv_PR3 F  Vv_PR3 R | GCAACCGATGTTGACATATCA  CGTCGCCCTAGCAAGTGAG | Chitinase 4c | Aziz et al., 2003  Trdá et al., 2014 |
| Vv_PAL F  Vv_PAL R | AGTCTCCATGGACAACACCCG  TGCTCAGCACTTTCGACATGG | Phenylalanine ammonia-lyase | Aziz et al., 2003  Dubreuil-Maurizi et al., 2010 |
| Vv_STS F  Vv_STS R | AGGAAGCAGCATTGAAGGCTC  TGCACCAGGCATTTCTACACC | Stilbene synthase | Trouvelot et al., 2008 |
| Vv_9-LOX F  Vv_9-LOX R | CTGGGTGGCTTCTGCTCTC  GCATGAATCTGCGGCTTATC | Lipoxygenase 9 | Dubreuil-Maurizi et al., 2010 |
| Vv_13-LOX F  Vv_13-LOX R | CATGGGTTGCTTCCAAGTTT  CAGGACTGCTGTCTGGATCA | Lipoxygenase 13 | (unpublished) |
| EF1 γ F  EF1 γ R | GAAGGTTGACCTCTGGGATG  AGAGCCTCTCCCTCAAAAGG | Elongation factor γ | Dufour et al., 2013 |
| EF1 α F  EF1 α R | GAACTGGGTGCTTGATAGGC  AACCAAAATATCCGGAGTAAAAGA | Elongation factor α | Dubreuil-Maurizi et al., 2010 |
